# High Gene Flow Despite Urbanization: Genetic Diversity and Structure of the Eastern Spotted Dove (*Spilopelia chinensis*) in Jiangsu Province, China and Implications for Releasing Confiscated Individuals

**DOI:** 10.1002/ece3.73966

**Published:** 2026-07-05

**Authors:** Dawei Liu, Gengdi Ying, Senlin Hou, Chunping Xie, Yalin Huang

**Affiliations:** ^1^ Faculty of Criminal Science & Technology Nanjing Police University Nanjing China; ^2^ Tropical Biodiversity and Bioresource Utilization Laboratory Qiongtai Normal University Haikou China

**Keywords:** Eastern Spotted Dove, gene flow, genetic diversity, mitochondrial DNA, population genetic structure, wildlife release

## Abstract

The Eastern Spotted Dove ( *Spilopelia chinensis* ) is a widespread human‐commensal bird, frequently involved in wildlife law enforcement cases. Following such cases, confiscated individuals are often released back into the wild, yet the potential genetic impacts of these releases remain unclear due to limited understanding of the species' population genetic structure in China. This study aimed to assess the genetic diversity and population structure of Eastern Spotted Doves in Jiangsu Province to provide a scientific basis for the management of confiscated individuals. This study analyzed the genetic diversity and structure of 139 individuals from 13 sampling sites across five avian geographical regions in Jiangsu Province, China, using mitochondrial cytochrome b (*Cytb*) and Displacement loop (*D‐loop*) sequences, and explored the impact of urbanization on genetic differentiation. A total of 49 haplotypes were detected. The samples exhibited high haplotype diversity (*H*_*d*_ = 0.896) but low nucleotide diversity (*pi* = 0.00216). The maximum likelihood phylogenetic tree showed that no geographical clustering, and the haplotype network displayed a star‐shaped topology with core haplotypes shared across all regions. AMOVA revealed that genetic differentiation among groups was negligible and non‐significant, regardless of grouping by geography or urbanization level. Pairwise *F*_*st*_ values were close to zero and non‐significant, while gene flow estimates (*N*_*m*_) exceeded 28 in all comparisons. These findings indicate that the Eastern Spotted Doves in Jiangsu Province lack significant population genetic structure, with strong gene flow maintained across regions. The species' high adaptability to urban environments and dispersal capacity likely sustain genetic connectivity. From a mitochondrial genetic perspective, the in situ or proximate release of confiscated individuals within Jiangsu Province poses minimal genetic contamination risk. However, validation using nuclear markers, such as microsatellites or single nucleotide polymorphisms, is recommended.

## Graphical abstract

This study reveals high haplotype diversity but low nucleotide diversity in Eastern Spotted Dove ( *Spilopelia chinensis* ) populations across Jiangsu Province, China, based on mitochondrial Cytb and D‐loop sequences from 139 individuals. Despite sampling from 13 sites spanning five geographical regions and varying urbanization levels, genetic analyses—including phylogenetic trees, haplotype networks, AMOVA, and pairwise FST—indicate negligible and non‐significant differentiation, with high gene flow (*N*_*m*_ > 14) maintaining genetic homogeneity. These results suggest strong dispersal and urban adaptability in the species, implying low risk of genetic contamination from in‐province release of confiscated individuals, though nuclear marker validation is advised.

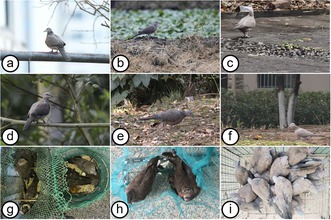

## 1 Introduction

**FIGURE 1:**
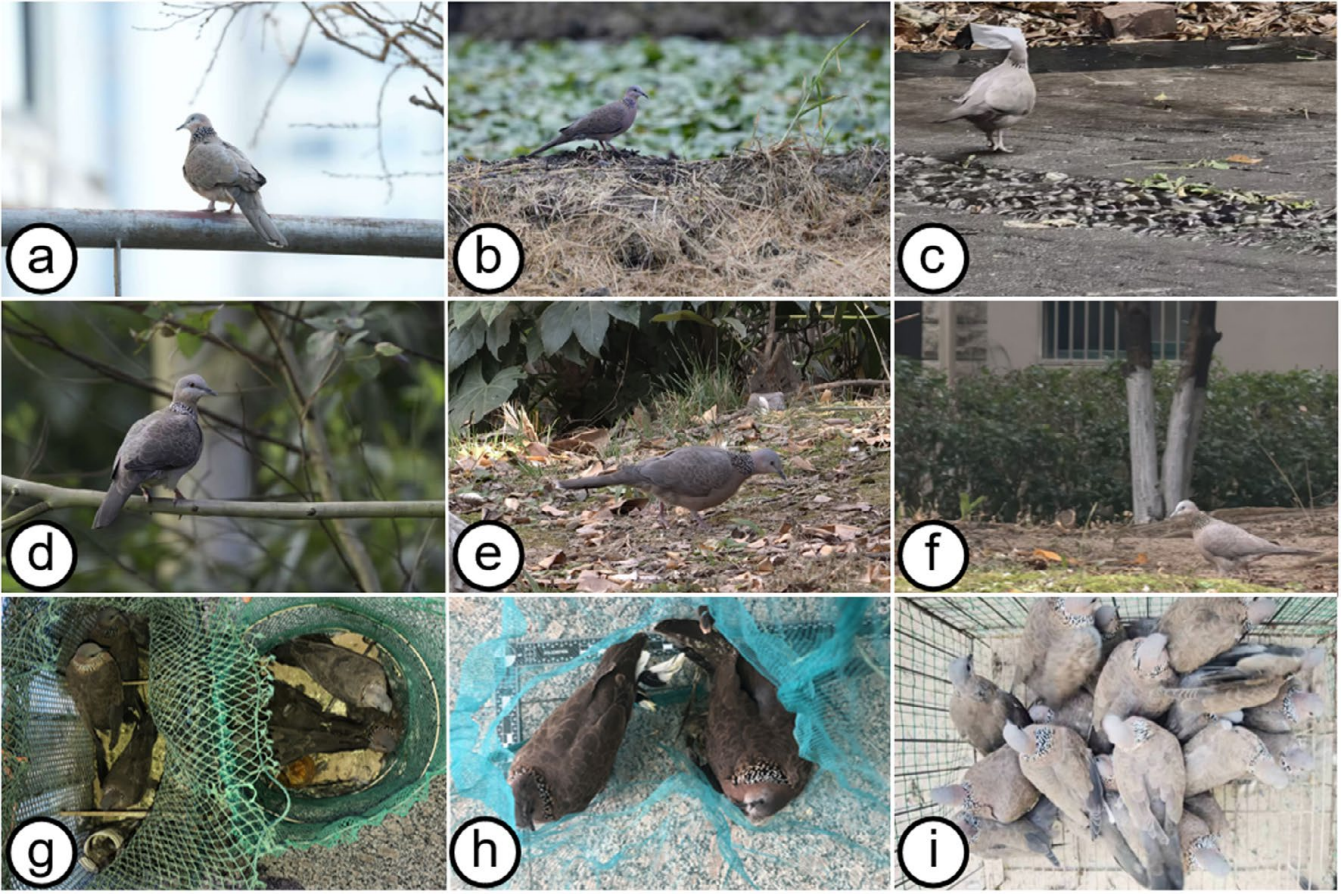
Photographs of the study species. (a) An Eastern Spotted Dove on a staircase handrail. (b) An Eastern Spotted Dove in farmland. (c) An Eastern Spotted Dove on a road. (d) An Eastern Spotted Dove in woodland. (e) An Eastern Spotted Dove in urban green space. (f) An Eastern Spotted Dove in a residential area. (g–i) Eastern Spotted Doves confiscated in wildlife law enforcement cases. Note: Variations in plumage coloration among images are due to differences in lighting conditions during photography.

The Eastern Spotted Dove ( *Spilopelia chinensis* ), a member of the family Columbidae within the order Columbiformes, is a common resident bird in East and South Asia, widely distributed across China (IUCN 2026). Across its range, the species is differentiated into five subspecies: *S. c. chinensis*, *S. c. suratensis*, *S. c. tigrina*, *S. c. hainana*, and *S. c. formosa* (Gill et al. 2025). Within China, three of these subspecies are recognized: *S. c. hainana* (restricted to Hainan Island), *S. c. tigrina* (occurring in Yunnan and southwestern Sichuan), and the widely distributed *S. c. chinensis*, which spans 28 province‐level administrative regions including Jiangsu (Zheng 2017). Beyond its native range, this adaptable dove has successfully colonized non‐native regions, establishing prominent invasive populations in Australia and Hawaii (Evans and Allmert 2026). Ecologically, this species exhibits high adaptability to various human‐modified habitats, ranging from less intensively managed areas such as farmland and suburban woodlands to highly urbanized environments such as parks and urban green spaces, and is frequently found around human settlements, making it a typical human‐commensal species (Figure 1； Yan and Ma 1994; Liu and Chen 2021).

In China, the Eastern Spotted Dove is included in the “List of Terrestrial Wild Animals with Important Ecological, Scientific, and Social Values”, a national list under China's Wildlife Protection Law that protects species from illegal hunting and trading, although they are not classified as nationally protected wildlife (Category I or II). As a result, confiscated individuals from illegal trade must be properly handled by wildlife authorities. Despite its protected status, its wide distribution, relatively large population size, and significant spatial overlap with human activities, combined with market demand for food consumption, make this species a frequent target of illegal trapping and trading (Figure 1; Liu et al. 2019; Hou 2020). Consequently, how to handle these confiscated individuals—particularly how to determine suitable release sites to avoid genetic pollution or maladaptation—has become an important practical issue in conservation and management. The key to addressing this issue lies in clarifying the species' population genetic structure—specifically, the spatial distribution of genetic variation and the degree of gene flow and isolation among populations (Slatkin 1985).

Inappropriate releases without consideration of population genetic structure may lead to negative consequences. For example, mixed‐source reintroductions of slimy sculpin ( *Cottus cognatus* ) resulted in outbreeding depression, with second‐generation hybrids showing reduced growth rate, length, and weight compared to pure strain individuals (Huff et al. 2011). In Europe, large‐scale releases of farmed mallards ( *Anas platyrhynchos* ) for hunting purposes have resulted in genetic admixture between released and wild populations, altering the genetic composition of wild populations and potentially posing unknown long‐term consequences for conservation (Söderquist et al. 2017). In China, wildlife release has a long history and has been increasing in recent years. However, many release activities lack proper genetic considerations, raising concerns about potential negative impacts on wild populations (Lin et al. 2023). Conversely, releasing individuals into populations that match their genetic background can effectively reduce genetic risks and improve release success rates (Fernandes and Caparroz 2013).

Notably, urbanization, as the most pronounced anthropogenic landscape transformation process worldwide, is profoundly reshaping wildlife habitats and evolutionary trajectories (Schmidt et al. 2020). In this process, natural habitats are increasingly encroached upon by urban development and transportation networks, leading to intensified habitat fragmentation (Liu et al. 2016). This may impede animal dispersal, increase spatial segregation between populations, and restrict gene flow. Such isolation can accelerate genetic drift, reduce effective population size, and ultimately lead to loss of genetic diversity and geographical genetic differentiation (Schlaepfer et al. 2018; Cueva et al. 2025). However, for species that are highly adapted to urban environments, such as the Eastern Spotted Dove, does urbanization similarly lead to genetic isolation among populations? Or can their strong dispersal capacity and adaptability to artificial habitats maintain gene flow between populations? These questions currently lack systematic investigations.

**FIGURE 2:**
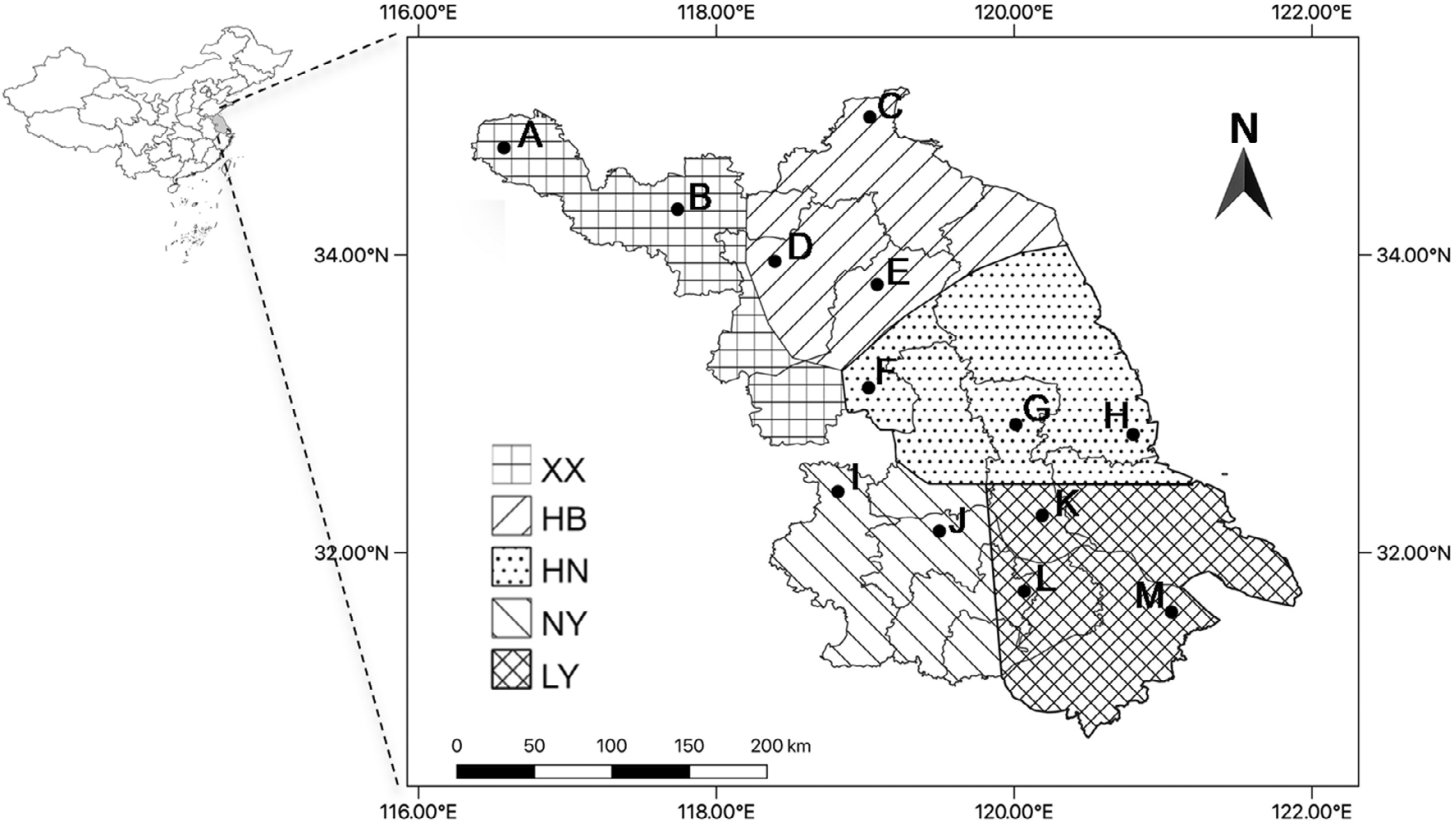
Locations of *Spilopelia chinensis* sampling across five avian geographical regions in Jiangsu Province, China.

To address these globally relevant questions, Jiangsu Province, a coastal province in eastern China, provides an ideal study area. The province can be divided into five avian geographical regions based on habitat types, climate, and vegetation: the Xuzhou‐Xuyi hilly and platform region (XX), the Huaibei plain region (HB), the Huainan plain region (HN), the Nanjing‐Yixing hilly and mountainous region (NY), and the Lower Yangtze plain region (LY) (Figure 2; Fei 2011). These five regions span from northwest to southeast, exhibiting a transition in topography from hilly uplands to alluvial plains and a gradual shift in climate from warm‐temperate semi‐humid monsoon to subtropical humid monsoon. Correspondingly, vegetation types form a continuous spectrum from warm‐temperate deciduous broad‐leaved forests in the northwest (XX and HB), through mixed deciduous‐evergreen broad‐leaved forests in the central transition zone (HN), to subtropical mixed deciduous‐evergreen and evergreen broad‐leaved forests in the southwest and southeast (NY and LY). Moreover, urbanization levels vary considerably across these regions, with the Lower Yangtze plain region being highly urbanized as part of the Yangtze River Delta metropolitan area, while the northern regions remain relatively less developed. This environmental heterogeneity, combined with varying urbanization intensities, makes Jiangsu Province a suitable model for examining whether landscape and anthropogenic factors influence the population genetic structure of the Eastern Spotted Dove.

In the present study, Eastern Spotted Dove samples were collected from 13 sampling sites across five avian geographical regions in Jiangsu Province, China. Based on sequences of the mitochondrial DNA (mtDNA) cytochrome b (*Cytb*) gene and the Displacement loop (*D‐loop*), this study aims to address the following questions: (1) What is the level of genetic diversity of Eastern Spotted Dove in Jiangsu Province? (2) Is there significant genetic differentiation among avian geographical regions, and what is the pattern of gene flow? (3) Does the level of urbanization affect the population genetic structure of the Eastern Spotted Dove? The results are intended to clarify the population genetic patterns of the Eastern Spotted Dove in Jiangsu Province and provide genetic information relevant to the release of confiscated individuals.

## 2 Materials and Methods

### 2.1 Sample Collection

The samples in this study were collected from the leg muscle of dead Eastern Spotted Doves seized by public security agencies in Jiangsu Province during the investigation of illegal wildlife trade cases. The capture locations of these individuals were documented at the time of confiscation. In total, 139 individuals were sampled across 13 distinct sites spanning the five avian aforementioned geographical regions (Figure 2). The samples were stored at −80°C until DNA extraction.

### 2.2 Urbanization Rate Data

Urbanization rate data for each region were obtained by consulting the statistical yearbooks of the cities where the samples were collected. Using an urbanization rate of 70% as the threshold, sampling sites located in cities with an urbanization rate ≥ 70% were classified as highly urbanized areas, while those in cities with a rate < 70% were classified as moderately urbanized areas. Among the 13 sampling sites, A–F and H were located in the moderately urbanized areas, whereas I–M and G were located in the highly urbanized areas (Table 1).

**TABLE 1:** Sampling information for *Spilopelia chinensis* in Jiangsu Province.

Avian geographical region	Sampling site	Number of samples (*n*)	City	Urbanization rate (%)^a^
XX	A	4	Xuzhou	67.64
	B	16	Xuzhou	67.64
HB	C	4	Lianyungang	64.00
	D	15	Suqian	65.00
	E	7	Huai'an	67.70
HN	F	8	Huai'an	67.70
	G	4	Taizhou	70.08
	H	8	Yancheng	66.27
NY	I	27	Nanjing	87.20
	J	3	Zhenjiang	80.70
LY	K	13	Taizhou	70.08
	L	10	Changzhou	78.01
	M	20	Suzhou	82.50

^a^ Urbanization rates were obtained from the statistical bureaus of respective cities: Xuzhou Municipal Bureau of Statistics and Xuzhou Investigation Team of the National Bureau of Statistics (2024), Lianyungang Municipal Bureau of Statistics and Lianyungang Investigation Team of the National Bureau of Statistics (2024), Suqian Municipal Bureau of Statistics and Suqian Investigation Team of the National Bureau of Statistics (2024), Huai'an Municipal Bureau of Statistics and Huai'an Investigation Team of the National Bureau of Statistics (2024), Taizhou Municipal Bureau of Statistics and Taizhou Investigation Team of the National Bureau of Statistics (2024), Yancheng Municipal Bureau of Statistics and Yancheng Investigation Team of the National Bureau of Statistics (2024), Nanjing Municipal Bureau of Statistics and Nanjing Investigation Team of the National Bureau of Statistics (2024), Zhenjiang Municipal Bureau of Statistics and Zhenjiang Investigation Team of the National Bureau of Statistics (2024), Changzhou Municipal Bureau of Statistics and Changzhou Investigation Team of the National Bureau of Statistics (2024), Suzhou Municipal Bureau of Statistics and Suzhou Investigation Team of the National Bureau of Statistics (2024).

### 2.3 DNA Extraction and Gene Amplification

Genomic DNA was extracted using the Universal Genomic DNA Extraction Kit (TaKaRa Biotech, Beijing, China). Primers for *Cytb* and *D‐loop* amplification were designed using Primer Premier 6.0 based on the complete mitochondrial genome sequence of the Eastern Spotted Dove (GenBank accession number: KP636801). Primer sequences are provided in Table 2. PCR reactions were performed in a total volume of 25 μL comprising the following components: 12.5 μL of Premix Taq (TaKaRa Biotech, Beijing, China), 1.5 μL of genomic DNA, 1 μL of each primer, and 9 μL of deionized water. Thermal cycling conditions were as follows: 95°C for 5 min; 35 cycles of 94°C for 30 s, 54°C for 35 s, and 72°C for 1 min; and a final extension at 72°C for 10 min. Following amplification, products were verified on a 1% agarose gel and sent for bidirectional sequencing to Sangon Biotech Company (Nanjing, China).

**TABLE 2:** Primer information.

Gene/Region	Primer name	Primer sequence (5′‐3′)	Length of target fragment (bp)
*Cytb*	Cytb_Dove F	CACATTACACCGCAGACA	905
	Cytb_Dove R	GGAAGAGGATAAGGAGGAT	
*D‐loop*	DL_Dove F	GCATTCGTGCCCTATGTACTAC	887
	DL_Dove R	CTTTACAGTGACTTGCGGAC	

### 2.4 Data Analysis

Sequences for each gene region were edited and aligned using SeqMan Pro v9 (DNAstar Inc.). The aligned sequences were then manually corrected in ChromasPro v2.1.3 (Technelysium Pty Ltd.). Genetic diversity indices were calculated for *Cytb* and *D‐loop* sequences both separately and concatenated. The concatenated sequences were used for phylogenetic analysis, haplotype network construction, and genetic structure analyses. Molecular genetic diversity indices—including the number of polymorphic sites (*S*), number of haplotypes (*H*), haplotype diversity (*H*_*d*_), nucleotide diversity (*pi*), and average number of nucleotide differences (*k*)—were calculated for each avian geographical region using DnaSP v5.0 with default settings (Librado and Rozas 2009). The best‐fit substitution model was selected using ModelFinder (Kalyaanamoorthy et al. 2017). Maximum likelihood phylogeny was inferred from concatenated sequences of each haplotype using IQ‐TREE v3.0.1 (Nguyen et al. 2015) with 5000 ultrafast bootstraps under the TN + G4 model. *Aratinga solstitialis* was used as an outgroup for rooting the tree. To visualize the connectedness and evolutionary pathways among haplotypes, a TCS Network was constructed from the mtDNA sequences of *S. chinensis* using PopART v1.7 with default settings (Leigh and Bryant 2015). To evaluate genetic structure, estimation of pairwise *F*_*st*_ (with 1000 permutations) and AMOVA were performed in Arlequin v3.5 (Excoffier and Lischer 2010), and gene flow (*N*_*m*_) was estimated based on *F*_*st*_ values. For the hierarchical AMOVA, samples were clustered into two groups based on the urbanization rate: the moderately urbanized cluster included sampling sites A–F and H, while the highly urbanized cluster comprised sampling sites I–M and G.

## 3 Results

### 3.1 Genetic Diversity Among Different Avian Geographical Regions

In this study, 32 haplotypes were detected in the *D‐loop* region, 26 haplotypes in the *Cytb* gene, and 49 haplotypes were identified from the concatenated sequences (Table 3). With the exception of the HB region, the genetic diversity of the *D‐loop* region was higher than that of the *Cytb* gene in the other four regions.

**TABLE 3:** Summary of the genetic diversity across avian geographical regions based on *D‐loop*, *Cytb*, and concatenated sequences.

Region	*D‐loop* sequence					*Cytb* sequence					Concatenated sequence				
	*S*	*H*	*H*_*d*_	*pi*	*k*	*S*	*H*	*H*_*d*_	*pi*	*k*	*S*	*H*	*H*_*d*_	*pi*	*k*
XX	24	9	0.753	0.00343	3.0158	23	8	0.795	0.00333	2.9789	47	11	0.905	0.00333	5.9053
HB	9	10	0.791	0.00167	1.4708	19	10	0.711	0.00233	2.0738	28	17	0.932	0.00209	3.6985
HN	8	8	0.774	0.00157	1.3842	5	6	0.684	0.00138	1.2316	13	10	0.863	0.00141	2.5053
NY	24	11	0.740	0.00280	2.4621	23	11	0.708	0.00258	2.3058	47	16	0.857	0.00265	4.6919
LY	19	16	0.809	0.00180	1.5814	8	9	0.536	0.00122	1.0875	27	21	0.911	0.00170	3.0066
Total	40	32	0.772	0.00219	1.9264	36	26	0.665	0.00204	1.8223	76	49	0.896	0.00216	3.8337

*Note:* *S* = number of polymorphic sites; *H* = number of haplotypes; *H*_*d*_ = haplotype diversity; *pi* = nucleotide diversity; *K* = average number of nucleotide differences.

The genetic diversity analysis based on the concatenated sequences showed that the overall *H*_*d*_ of Eastern Spotted Doves in Jiangsu Province was 0.896, indicating a relatively high level. The overall *S*, *pi*, and *k* were 76, 0.00216, and 3.8337, respectively. Differences were observed among avian geographical regions: the XX region had the highest nucleotide diversity (*pi* = 0.00333); the HB region showed the highest haplotype diversity (*H*_*d*_ = 0.932); and the LY region had the highest number of haplotypes (*H* = 21; Table 3).

### 3.2 Haplotype Diversity and Phylogenetic Relationships

**FIGURE 3:**
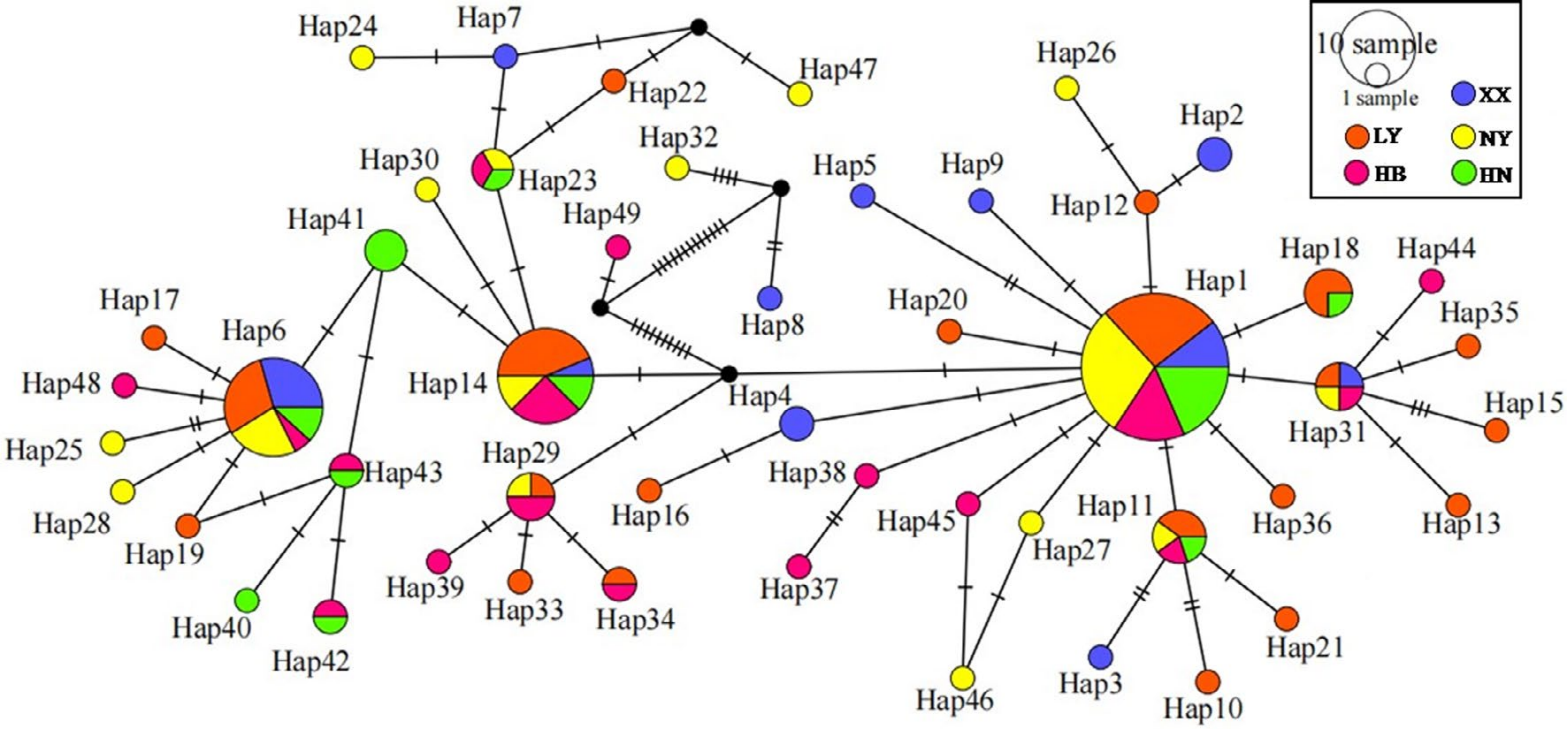
Haplotype network of 49 concatenated haplotypes. The area of each node corresponds to its haplotype frequency. Unsampled or inferred intermediate haplotypes are represented by black dots (median vectors).

The median‐joining network constructed from the concatenated sequences revealed the presence of three high‐frequency core haplotypes (Hap 1, Hap 6, and Hap 14) (Figure 3). These three haplotypes were shared across all five avian geographical regions, and their individual frequencies were significantly higher than those of other haplotypes. Furthermore, Hap 11 and Hap 31 were shared by four regions; Hap 23 and Hap 29 were shared by three regions; and the remaining haplotypes were distributed in only one or two regions.

**FIGURE 4:**
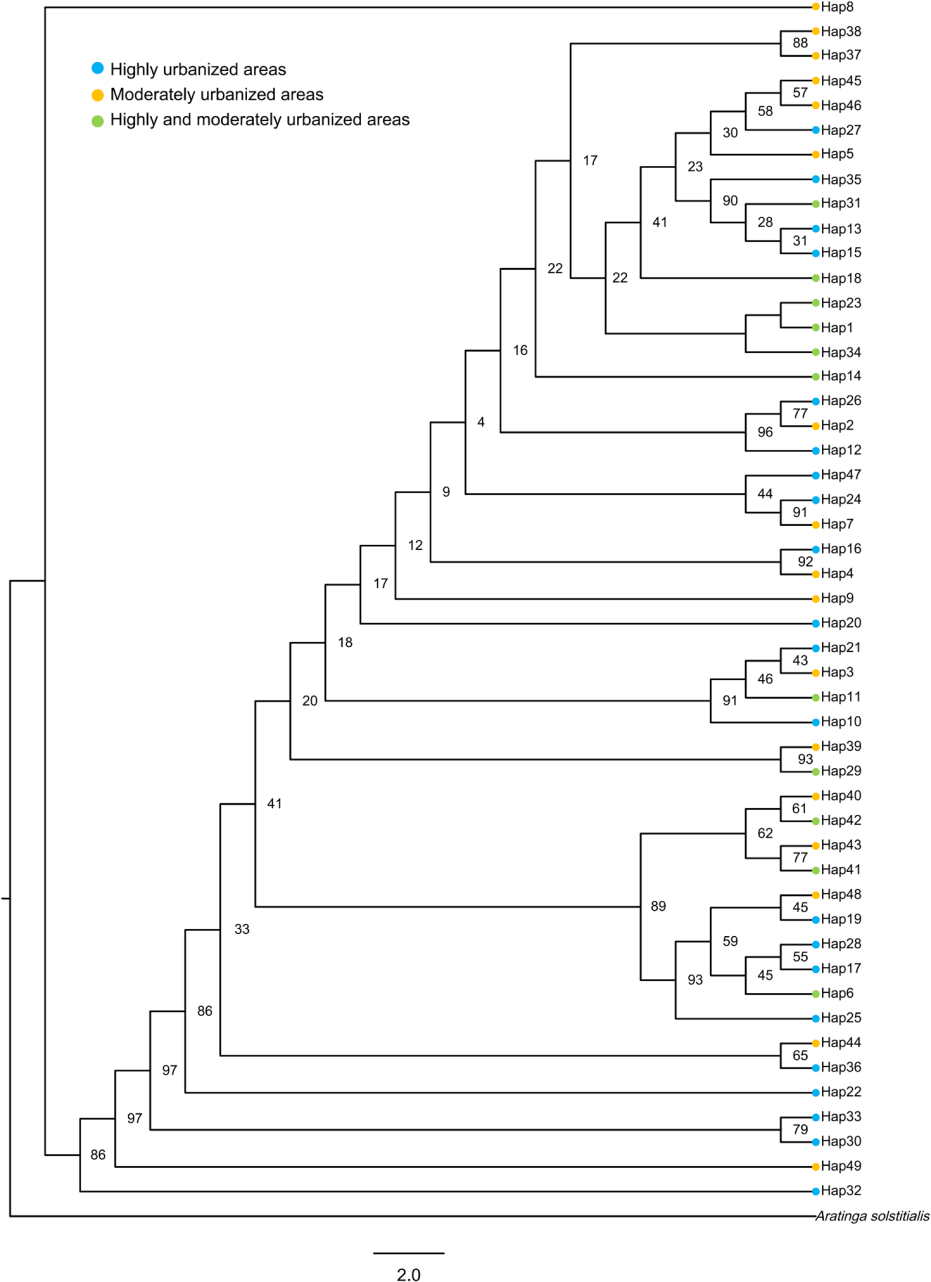
Maximum likelihood phylogenetic tree based on mitochondrial concatenated sequence haplotypes. Numbers at nodes indicate bootstrap support values. Colors of circles indicate the urbanization level corresponding to each haplotype: blue = highly urbanized areas, yellow = moderately urbanized areas, green = haplotypes found in both highly and moderately urbanized areas. *Aratinga solstitialis* was used as the outgroup.

Haplotypes from highly urbanized and moderately urbanized areas were intermingled throughout the tree, with no clustering by urbanization level (Figure 4). Bootstrap support values varied considerably across the phylogenetic tree. Some terminal clades received relatively high support (e.g., 91% for Hap 24‐Hap 7, 92% for Hap 16‐Hap 4, 93% for Hap 39‐Hap 29), while most deeper nodes received low support (< 50%), reflecting limited phylogenetic signal at the intraspecific level.

### 3.3 Genetic Structure and Gene Flow

To examine whether urbanization level influences the genetic structure of Eastern Spotted Doves, we performed AMOVA, which partitions genetic variation between predefined hierarchical levels. The results showed that when all samples were divided into two groups—highly urbanized areas and moderately urbanized areas—the between‐group genetic differentiation index (*F*_*ct*_ = −0.00754) was close to zero and statistically non‐significant (*p* > 0.05; Table 4). This indicated no significant genetic differentiation between samples from highly urbanized and moderately urbanized areas, suggesting that urbanization level did not significantly affect the genetic structure. Consistently, the percentage of genetic variation between urbanization groups was negligible (−0.75%), with the majority of variation (99.74%) found within avian geographical regions.

**TABLE 4:** AMOVA results for concatenated mtDNA sequences of *Spilopelia chinensis* grouped by urbanization level.

Source of variation	Df	Sum of squares	Percentage variation	*F*‐statistics	Value	*p*
Between groups	1	0.279	−0.75	*F*_*ct*_	−0.00754	0.92669
Among avian geographical regions within groups	11	5.398	1.01	*F*_*sc*_	0.00257	0.21310
Within avian geographical regions	126	56.165	99.74	*F*_*st*_	0.00257	0.28055

Pairwise *F*_*st*_ values among avian geographical regions ranged from −0.01209 to 0.01754, none of which reached statistical significance (*p* > 0.05; Table 5). The *N*_*m*_ values among all avian geographical regions exceed 28, with values approaching infinity for some pairwise comparisons. This indicated an extremely low level of genetic differentiation and an absence of population structure among regions.

**TABLE 5:** Values of genetic differentiation (*F*_*st*_, below the diagonal) and gene flow (*N*_*m*_, above the diagonal) among avian geographical regions based on the concatenated mtDNA sequences.

	XX	HB	HN	NY	LY
XX		29.368	28.006	59.452	67.900
HB	0.01674		∞	2499.500	∞
HN	0.01754	−0.00651		∞	∞
NY	0.00834	0.00020	−0.01209		166.417
LY	0.00731	−0.01127	−0.00342	0.00150	

*Note:* *F*_*st*_ = genetic differences; *N*_*m*_ = gene flow; ∞ = infinite gene flow (when *F*_*st*_ ≤ 0).

## 4 Discussion

### 4.1 Genetic Diversity of *S. chinensis* in Jiangsu Province

In this study, we employed two mitochondrial markers to provide a comprehensive view of the genetic diversity of the Eastern Spotted Dove (Sun et al. 2023; Chen et al. 2026). Based on these concatenated sequences, our analysis of 139 Eastern Spotted Doves from 13 locations in Jiangsu Province revealed a high level of mitochondrial genetic diversity (*H*_*d*_ = 0.896, *pi* = 0.00216). This finding aligns with studies on other widespread bird species, such as the Light‐vented Bulbul ( *Pycnonotus sinensis* ) (*H*_*d*_ = 0.995, *pi* = 0.0054; Song et al. 2013), showing that human‐associated birds often exhibit high haplotype diversity.

Genetic diversity serves as the foundation for a species' adaptation to future environmental changes (Frankham 2005; Hughes et al. 2008). The high mitochondrial genetic diversity observed in this study suggests that the Eastern Spotted Dove population in Jiangsu Province may have a favorable basis for maintaining stable numbers in rapidly changing human‐dominated landscapes.

This study also found that the samples exhibit a “high haplotype diversity– low nucleotide diversity” genetic pattern, which is often interpreted as a genetic signature of recent rapid population expansion (Zhang et al. 2020). This occurs because new haplotypes emerge rapidly during an expansion, but insufficient time has passed for substantial nucleotide divergence to accumulate (Grant and Bowen 1998). The Eastern Spotted Dove, as a typical human‐commensal species, effectively utilizes food and habitat resources in urban environments (Tang et al. 2018; Ramirez et al. 2025). Its population expansion is likely closely linked to its high environmental adaptability (Menon 2025).

### 4.2 Genetic Structure and Gene Flow Patterns

An important finding of this study is that the Eastern Spotted Doves in Jiangsu Province exhibit no significant genetic differentiation among avian geographical regions. This conclusion is supported by multiple lines of evidence: the haplotype network shows that high‐frequency core haplotypes are shared across all five avian geographical regions. Meanwhile, pairwise *F*_*st*_ values between geographical regions are all close to zero, and *N*_*m*_ values all exceed 28—with some even approaching infinity—quantitatively confirming extremely active gene exchange (Wright 1931; Slatkin 1985).

This pattern of genetic connectivity and strong gene flow is likely maintained by a combination of the landscape characteristics of Jiangsu Province (flat terrain with no geographic barriers) and the species' biological traits, including its strong dispersal ability and high adaptability to human‐modified environments. Major geographical barriers such as mountain ranges are important factors limiting bird dispersal and gene flow (White 2016; Machado et al. 2018). In China, the Yanshan Mountains, for example, have been shown to act as a significant north–south phylogeographic barrier for three eastern forest‐dwelling bird species (Song et al. 2016). In contrast, Jiangsu Province is characterized by flat terrain dominated by plains and low hills, lacking such natural geographical barriers. This topographical feature theoretically provides favorable physical conditions for unrestricted bird dispersal. Furthermore, pigeons (Columbidae) are recognized as a highly dispersive family (Sheard et al. 2020). As a columbid, the Eastern Spotted Dove is expected to possess strong flight capability. Such dispersal ability allows individuals to easily cross landscape elements such as urban blocks, farmlands, and rivers, thereby facilitating gene flow among geographical regions (Canales‐Delgadillo et al. 2012).

### 4.3 Impact of Urbanization on Genetic Structure

Urbanization is often regarded as a key driver that promotes genetic differentiation among groups by fragmenting habitats and restricting gene flow (Johnson and Munshi‐South 2017). However, this study indicates that the level of urbanization did not significantly affect the genetic structure of Eastern Spotted Doves in Jiangsu Province. AMOVA revealed that the *F*_*ct*_ value between highly and moderately urbanized areas was close to zero and non‐significant, meaning samples from the two types of areas were genetically indistinguishable. This finding supports the view that the impact of urbanization on wildlife genetic structure is not uniform but exhibits considerable species‐specific variation. For example, Indykiewicz et al. (2018) reported that urbanization did not act as an effective barrier to gene flow in Black‐headed Gulls ( *Chroicocephalus ridibundus* ), with sufficient genetic exchange occurring between urban and rural populations. Similarly, Carlen and Munshi‐South (2021) found that feral pigeons ( *Columba livia* ) in six major cities in the northeastern United States maintained extensive gene flow and very low genetic differentiation despite high levels of urbanization.

Beyond the factors discussed above, the absence of genetic differentiation along the urbanization gradient is also attributable to the species' ecological habits and urban landscape structure. This species exhibits high adaptability to urban environments, enabling it to extensively utilize artificial habitats such as urban green spaces, parks, and residential areas. As a typical human‐commensal species, it can exploit various resources while completing its life cycle in diverse habitats. Such adaptability reduces the species' reliance on specific continuous pristine habitats (Rodríguez‐Bardía et al. 2022). Furthermore, although urbanization intensifies the fragmentation of natural habitats, the dense urban green space systems, extensive farmland networks, and crisscrossing river corridors in Jiangsu Province collectively form a functional network of greenways and stepping stones that connect different habitat patches (Lynch 2018). These linear and patchy habitats likely provide gradual dispersal pathways for Eastern Spotted Doves, thereby mitigating the potential population isolation caused by habitat fragmentation and maintaining a high level of gene flow between urban and rural populations (Hambuckers et al. 2023; Suraci et al. 2023).

However, it should be noted that the highly urbanized sites in this study were predominantly located in southern Jiangsu Province, while moderately urbanized sites were mainly distributed in northern regions. This spatial distribution may introduce a potential confounding effect between urbanization level and north–south geography. Nevertheless, given that neither the AMOVA between urbanization groups (*F*_*ct*_ = −0.00754, *p* > 0.05) nor the pairwise *F*_*st*_ values among avian geographical regions showed significant genetic differentiation, it appears unlikely that either urbanization or geographic location has substantially influenced the genetic structure of Eastern Spotted Doves in the study area.

### 4.4 Implications for the Release of Confiscated Individuals

An important applied objective of this study is to provide preliminary reference for the science‐based release of confiscated Eastern Spotted Doves. Analysis based on mtDNA markers revealed that Eastern Spotted Doves from different avian geographical regions within Jiangsu Province exhibited no significant mitochondrial genetic structure, with no significant geographical genetic differentiation observed. From a conservation genetics perspective, these findings preliminarily suggest that in situ or proximate release of confiscated individuals within Jiangsu Province may not pose a significant genetic risk.

However, while these results provide valuable baseline data for immediate management decisions, it is important to emphasize that mitochondrial DNA, as a maternally inherited genetic marker, has inherent limitations. This marker only reflects the history of maternal gene flow and cannot capture patterns of gene exchange mediated by males (Zink and Barrowclough 2008). If Eastern Spotted Doves exhibit sex‐biased dispersal (i.e., female dispersal and male philopatry), mitochondrial markers may overestimate the actual level of gene flow relative to the nuclear genome. Additionally, the effective population size of mitochondrial DNA is only one‐quarter of that of the nuclear genome, making it more sensitive to genetic drift and, in certain scenarios, unable to accurately reflect the overall genetic structure of populations (Ballard and Whitlock 2004). In practical conservation genetics, genetic assessments involving reintroduction decisions generally recommend the combined use of both mitochondrial and nuclear genetic markers (Toews and Brelsford 2012; Domínguez et al. 2017).

In the absence of validation from nuclear genetic markers, it is recommended to use the findings of this study as a reference and to prioritize release sites with habitat conditions similar to those of the seizure locations when releasing confiscated Eastern Spotted Doves within Jiangsu Province. For individuals potentially originating from outside Jiangsu Province, a careful assessment remains necessary. Furthermore, genetic evaluation constitutes only one of the considerations in release decision‐making; ecological factors such as habitat quality and food resources are equally critical and should not be overlooked (IUCN/SSC 2013).

This study demonstrates that Eastern Spotted Doves in Jiangsu Province exhibit high mitochondrial genetic diversity and no significant genetic structure among avian geographical regions or between urbanization levels. These findings suggest that confiscated individuals could potentially be released in situ or at proximate locations within the province. However, given the inherent limitations of mitochondrial DNA as a maternally inherited marker, these conclusions require further validation using nuclear genetic markers, such as microsatellites or single nucleotide polymorphisms.

## Author Contributions

**Dawei Liu:** conceptualization (equal), methodology (lead), writing – original draft (lead), writing – review and editing (lead). **Gengdi Ying:** data curation (lead), formal analysis (lead), investigation (lead), software (lead), validation (lead). **Senlin Hou:** project administration (lead), visualization (lead). **Chunping Xie:** supervision (lead), writing – review and editing (supporting). **Yalin Huang:** conceptualization (equal), funding acquisition (lead), resources (lead).

## Funding

This study was supported by the Jiangsu Province “14th Five‐Year Plan” Key Construction Discipline “Public Security Technology” project (2022).

## Ethics Statement

All samples used in this study were obtained from deceased Eastern Spotted Doves confiscated by public security agencies during wildlife law enforcement operations. No live animals were captured or sacrificed for this research; therefore, no animal ethics approval was required.

## Conflicts of Interest

The authors declare no conflicts of interest.

## Data Availability

All raw data have been deposited in Science Data Bank. The data can be accessed via the following link: https://www.scidb.cn/s/Yn2Qze.
